# Editorial: Latest advancements in organ-on-a-chip technology

**DOI:** 10.3389/fphar.2025.1631320

**Published:** 2025-05-30

**Authors:** Jareer Kassis, Zhengpeng Wan

**Affiliations:** ^1^ Wake Forest Institute for Regenerative Medicine, Wake Forest University School of Medicine, Winston-Salem, NC, United States; ^2^ Department of Biological Engineering, Massachusetts Institute of Technology, Cambridge, MA, United States

**Keywords:** organoids, liver-on-a-chip, bone marrow, feto-maternal interface, osteoarthiritis, drug development

The importance of organ-on-a-chip technologies has been steadily rising as advances in their designs and implementations have rendered them increasingly valuable for simulating live organ function and understanding disease etiology. The ability to evaluate cellular systems in three-dimensional environments that resemble the milieus of their physiological counterparts has been a long-pursued goal that requires the coalescence of biology and engineering expertise. The goal is to produce robust systems that can help answer questions that remain unsolvable using traditional culturing methods and to realize the vision of replacing animal models with validated human cell-utilizing devices. To that end, efforts have focused on the ability to coculture various types of cells that comprise the simulated tissue of interest in dedicated chambers that allow for controlled nutrient circulation and waste removal while simultaneously enabling microscopic visualization, addition of agents, and periodic sampling of the system’s components (Bhatia and Ingber, 2014; Ronaldson-Bouchard and Vunjak-Novakovic, 2018).

This Research Topic describes some of the most recent advances in organ-on-a-chip technology that include systems designed to replicate human joints, the fetal-maternal interface, and bone marrow. Also included is a review of the current advances and challenges in studying drug metabolism using liver constructs.

Mirazi and Wood describe a practical model in which they aimed to replicate the environment of a human joint with which to study the pathogenesis of osteoarthritis. Their organ-on-a-chip system involves the coculturing of osteoblasts, chondrocytes, fibroblasts, and either quiescent or pro-inflammatory macrophages (to simulate healthy and diseased joints, respectively) in separate chambers through which common media flows sequentially, thereby establishing paracrine signaling. They validated the system’s integrity by testing for cell viability, metabolic activity, and membrane integrity. The authors state that the novelty of their device is in its handling of up to four joint-related cell types whereas previous models did not surpass two; this more closely reflects physiologic conditions.

Kammala et al. sought to address a critical drawback in drug testing during the development of therapeutics: the inability to perform studies on pregnant women given that pregnancy is a common clinical trial exclusion criterion. They established a multi-organ-simulating platform that included fetal membrane amniotic epithelial cells, amnion mesenchymal cells, chorion trophoblasts, maternal decidual cells, syncytiotrophoblast (using placenta-syncytialized cytotrophoblasts), cytotrophoblasts, and human umbilical vein endothelial cells; these constituted both the fetal and placental components of the feto-maternal interface (FMI). Their study employed the antioxidant drug pravastatin, a cholesterol-lowering agent that has also been used to treat preeclampsia (Mészáros et al., 2023). They successfully determined the metabolism of this drug as it crossed both FMI components and confirmed that its pharmacokinetics as measured in their system were commensurate with separately calculated in-silico projections.

Ritter et al. developed an *in vitro* model for the long-term cultivation of primary cell cultures using a background of bone marrow extracellular matrix. They validated this miniaturized optically accessible bioreactor (MOAB) with CD4^+^ T lymphocytes and cord blood-derived hematopoietic CD34^+^ cells, some of which survived for up to 3 months. Furthermore, they investigated whether the MOAB could detect emerging tumorigenic events given that cells cultured in body-on-a-chip systems that are undergoing gene correction can become transformed as a result of such interventions; they were able to pinpoint as few as 100 leukemic cells in a sea of 100,000 primary cells. This helped establish their MOAB as a potential detector of cellular transformation during long-term three-dimensional culturing.

Lastly, a review by Tamargo-Rubio et al. details the current state of knowledge around using liver-on-a-chip systems for studying drug metabolism *ex vivo*. The authors propose that human induced pluripotent stem cells would be ideal, as they can differentiate into the organ of interest while preserving donor-specific phenotypes, thereby allowing for exploring the metabolism of specific drugs in patient-derived liver cells before deciding on optimal treatment. They specifically discuss ideas for improving the expression of CYP450 in liver-on-a-chip systems (given this enzyme’s importance in drug metabolism); such ideas include increasing tissue complexity by growing organoids in optimal matrices before their incorporation into the organ-on-a-chip, controlling the nature of growth factors used during pluripotent stem cell differentiation in media, and allowing such cells to differentiate in the presence of other liver cell types to promote greater CYP450 expression.

The articles included in this Research Topic clearly illustrate the leaps forward in complexity and applicability that researchers continue to achieve in advancing organ-on-a-chip technology. Such progress comes as the United States Food and Drug Administration has announced plans to phase out animal testing for monoclonal antibodies and certain other agents in favor of alternative technologies. The suggested substitutes include human organoid-based laboratory models, which the Administration deems more sensitive and pertinent for toxicological and other tests than non-human species (Food and Drug Administration, 2025). This is in part a testament to the success of organoid/organ-on-a-chip systems and their ability to simulate physiologic environments with a high degree of accuracy.
